# Long Noncoding RNA HCG18 Promotes Malignant Phenotypes of Breast Cancer Cells *via* the HCG18/miR-103a-3p/UBE2O/mTORC1/HIF-1α–Positive Feedback Loop

**DOI:** 10.3389/fcell.2021.675082

**Published:** 2021-12-07

**Authors:** Xu Liu, Kun Qiao, Kaiyuan Zhu, Xianglan Li, Chunbo Zhao, Jiaqi Li, Dawei Feng, Yu Fang, Peng Wang, Cheng Qian, Wenbo Qiao

**Affiliations:** ^1^ Department of Abdominal Radiotherapy, Harbin Medical University Cancer Hospital, Harbin Medical University, Harbin, China; ^2^ Department of Breast Surgery, Harbin Medical University Cancer Hospital, Harbin Medical University, Harbin, China; ^3^ Department of Radiotherapy Technology Center, Harbin Medical University Cancer Hospital, Harbin Medical University, Harbin, China; ^4^ Department of Oncology Phase I Clinical Research, Harbin Medical University Cancer Hospital, Harbin Medical University, Harbin, China

**Keywords:** HCG18, UBE2O, MiR-103a-3p, breast cancer, mechanistic target of rapamycin complex 1

## Abstract

In recent years, an increasing number of studies have reported that long noncoding RNAs (lncRNAs) play crucial roles in breast cancer (BC) progression and metastasis. Another study group of our research center reported that lncRNA HCG18 was one of the 30 upregulated lncRNAs in BC tissues compared with normal tissues in The Cancer Genome Atlas database. However, the exact biological roles of HCG18 in BC remain unclear. In this study, we demonstrated that HCG18 is significantly upregulated in BC tissues and cells and that BC patients with high HCG18 expression tend to have poor prognosis. *In vitro* assays indicated that HCG18 promotes BC cell proliferation and invasion and endows BC cells with cancer stemness properties. *In vivo* assays revealed that reducing HCG18 expression in the BC cell line MDA-MB-231 markedly decreased tumor growth and lung metastasis in xenograft mouse models. In terms of mechanism, we found that HCG18 positively regulated the expression of BC-related ubiquitin-conjugating enzyme E2O (UBE2O) by sponging miR-103a-3p, and our previous research verified that UBE2O could promote the malignant phenotypes of BC cells through the UBE2O/AMPKα2/mTORC1 axis. Furthermore, as a downstream target of the HCG18/miR-103a-3p/UBE2O/mTORC1 axis, hypoxia-inducible factor 1α transcriptionally promoted HCG18 expression and then formed a positive feedback loop in BC. Taken together, these results confirm that HCG18 plays an oncogenic role in BC and might serve as a prognostic biomarker and a potential therapeutic target for BC treatment.

## Background

Although early detection strategies, high-quality prevention strategies, and advanced therapeutic strategies have been applied in clinical practice, breast cancer (BC) is still the most common female cancer (30%) and the leading cause of cancer-related death (15%) worldwide (Siegel et al., 2021). The high rates of recurrence and metastasis are the main causes of the high mortality rate in BC. It has been reported that the overall 5-year survival rate of patients with early-stage BC is 92.5 to 85.9%; however, it is decreased to 25.1% for patients in stage IV disease (Wang et al., 2019). Therefore, promising detection markers and effective therapeutic methods are urgently needed in BC.

LncRNAs are a class of RNA transcripts that are longer than 200 nucleotides in length and have no or limited protein-coding capability (Wilusz et al., 2009). Recent studies have confirmed that lncRNAs are involved in a variety of physiological and pathological processes of human diseases, especially cancers (Cabili et al., 2011; Geisler, 2013). For example, high expression of Linc00941 promotes colorectal cancer metastasis by preventing SMAD4 protein degradation and activating the TGF-β/SMAD2/3 signaling pathway (Wu et al., 2021). LncRNA CASC9 promotes esophageal squamous cell carcinoma metastasis by upregulating LAMC2 expression by interacting with the CREB-binding protein (Liang et al., 2018). In addition, lncRNAs can function as competing endogenous RNAs (ceRNAs) by competitively adsorbing microRNAs (miRNAs), resulting in the loss or reduction of miRNA function and controlling the regulation of target genes by miRNAs. For instance, lncRNA PVT1 promotes gemcitabine resistance in pancreatic cancer by modulating the miR-619-5p/Pygo2 and ATG14 axes (Zhou et al., 2020). LncRNA HCP5 induces gastric cancer progression *via* the miR-186-5p/WNT5A axis (Gao et al., 2021). LncRNA HLA complex group 18 (HCG18) is characterized as a tumor-promoting lncRNA that plays critical roles in diverse human cancers. HCG18 promotes gastric cancer proliferation by adsorbing miR-141-3p and miR-197-3p (Liu et al., 2020a; Niu et al., 2020). HCG18 contributes to the progression of lung adenocarcinoma through the miR-34a-5p/HMMR axis (Li et al., 2020a). HCG18 could also accelerate colorectal cancer invasion in a miR-1271/MTDH/Wnt/β-catenin–dependent manner (Li et al., 2020b). Another group from our research center identified HCG18 as one of the 30 upregulated lncRNAs in BC by analyzing data from two cohorts in The Cancer Genome Atlas (TCGA) (Xu et al., 2017). However, its biological function in BC remains unclear.

Ubiquitin-conjugating enzyme E2O (UBE2O) is a large E2-ubiquitin-conjugation enzyme that has both E2 and E3 activities (Berleth, 1996). Abnormal expression of UBE2O exists in many types of human cancers, and deregulation of UBE2O plays crucial roles in tumor progression and metastasis (Lin et al., 2006; Rice et al., 2011; Toffoli et al., 2014). For a previous study, we performed an in-depth investigation of UBE2O and demonstrated that UBE2O could promote BC cell proliferation and epithelial–mesenchymal transformation (EMT) and endow BC cells with cancer stemness properties (CSPs) *via* the UBE2O/AMPKα2/mTORC1-MYC positive feedback loop (Liu et al., 2020b). However, the exact regulatory mechanism of UBE2O in BC still needs to be further investigated.

In this study, we confirmed that HCG18 is significantly upregulated in BC patients and has a close relationship with a poor prognosis in BC patients. HCG18 could promote BC cell proliferation and invasion, endow BC cells with CSPs *in vitro*, and facilitate tumor growth and metastasis *in vivo*. In terms of mechanism, we found that HCG18 could competitively adsorb miR-103a-3p, indirectly facilitating UBE2O expression and thus activating the UBE2O/mTORC1 axis in BC cells. Furthermore, as a downstream target of mTORC1, hypoxia-inducible factor 1α (HIF-1α) could transcriptionally promote HCG18 expression, forming a positive feedback loop in BC.

## Materials and Methods

### BC Cells and Specimens

All BC cells and normal mammary cells (MCF-10A) were purchased from the Cell Bank of the Chinese Academy of Sciences (Shanghai, China) and cultured according to the supplier’s instructions. Fresh BC tissues, adjacent noncancerous tissues, and corresponding paraffin-embedded cancer tissues (*n* = 120) were obtained from Harbin Medical University Cancer Hospital between 2012 and 2013. All the patients enrolled in our study had complete clinicopathological information, and patients who received neoadjuvant chemotherapy, radiotherapy, and immunotherapy and those with recurrent tumors, bilateral BC, or other previous tumors were excluded. Our study was approved by the Research Ethics Committee of Harbin Medical University, and all the patients enrolled in our study signed informed consent forms.

### RNA Extraction and Quantitative Real-Time PCR

Total RNA was extracted from fresh BC tissues and cells with TRIzol reagent (Invitrogen, United States) according to the manufacturer’s instructions. The Prime Script RT Reagent Kit with gDNA Eraser (Takara, Kyoto, Japan) was used to synthesize cDNA, and qRT-PCR was conducted using SYBR Premix Ex Taq™ II (Takara) on a CFX96 Touch Detection System (Bio-Rad, United States). The primers used in our study are listed in Supplementary Table S1. GAPDH was used as an internal control for detecting mRNA expression; LncRNA (HCG18) expression was normalized by 18S rRNA and miRNA expression were normalized by U6. The relative RNA abundance was calculated by the standard 2^−∆∆CT^ method.

### Protein Expression Analysis

Western blotting assays were performed as previously reported (Liu et al., 2020b). Briefly, the indicated cells were lysed with RIPA (Beyotime, China) buffer containing phenylmethylsulfonyl fluoride (Beyotime). Then, the lysate was collected, and the proteins were separated by 10% sodium dodecyl sulfate–polyacrylamide gel electrophoresis and transferred to polyvinylidene fluoride membranes. After that, the membranes were incubated with primary antibodies and secondary antibodies. Finally, the protein bands were detected using a ChemiDoc Touch imaging system (Bio-Rad). The antibodies used are listed in Supplementary Table S2.

### Cell Proliferation Assay

For the Cell Counting Kit-8 (CCK-8) assays, the indicated cells were seeded into 96-well plates at a density of 2 × 10^3^ cells per well. At each preset time, 10 μL CCK-8 reagent (Beyotime) was added to each well containing 90 μL culture medium, and the plates were incubated at 37°C for 2 h. After that, the cell viability was detected by measuring the absorbance at 450 nm.

For the colony formation assays, the indicated cells were seeded into six-well plates (5 × 10^2^ cells/well) and incubated with culture medium containing 10% fetal bovine serum (FBS) at 37°C for 2 weeks. After that, the cells were fixed with formalin for 30 min and stained with crystal violet. A FluorChem M system was used to take the photographs.

5-Ethynyl-2′-deoxyuridine (EdU) staining assays were performed as follows: the indicated cells were seeded into 96-well plates and cultured with 10% FBS medium at 37°C. After the confluence reached 60–70%, the cells were subjected to EdU staining (Beyotime) according to the manufacturer’s instructions. After fixation and permeabilization, the cells were stained with EdU and DAPI solutions. The samples were photographed and analyzed under a fluorescence microscope.

### Scratch Assays and Cell Invasion Assays

For wound scratch assays, the indicated cells were seeded into six-well plates and cultured at 37°C. After the cells reached 95% confluence, sterile micropipette tips were used to make scratch wounds. Then, the cells were washed with PBS and cultured with serum-free medium continuously. Images were taken under a microscope at preset times, and the migration rate was subsequently analyzed. The percentage of wound closure (%) = (width on 0 h—with on 24 or 48 h)/width on 0 h × 100%.

For cell invasion assays, the upper chambers of 24-well Transwell plates (8.0 µm) (Corning, United States) were coated with Matrigel and incubated at 37°C for 4 h. Then, 200 μL serum-free medium containing the indicated cells (1 × 10^5^) was added to the upper chambers of the Transwell plates, and 600 μl 10% FBS medium was added to the lower chambers of the Transwell plates. Then, the cells were cultured at 37°C for 24 h. Afterward, the noninvaded cells were scraped from the upper side of the chambers, and invaded cells on the lower side were fixed with 4% paraformaldehyde and stained with 1% crystal violet. Finally, the images were captured, and the invaded cells were counted under a microscope.

### Hematoxylin–Eosin Staining and Immunohistochemistry

Hematoxylin–eosin (HE) staining assays were performed according to a standard HE staining technique as previously reported (Chong and Murphy, 2004). Briefly, mouse lung tissues were fixed in 4% paraformaldehyde, embedded in paraffin, and sectioned (5-mm thickness). After that, the lung sections were subjected to HE staining to evaluate pathological changes and photographed under an optical microscope.

Immunohistochemistry (IHC) staining was performed on paraffin-embedded specimens from BC patients following a standard streptavidin–peroxidase complex procedure. The staining results were evaluated and calculated as histochemistry scores (*H* scores) by three senior pathologists independently. The following antibodies were used: anti-UBE2O (catalog no. 15812-1-AP; Proteintech Group, China) and anti–Ki-67 (catalog no. 9449; CST, United States).

### Sphere Culture and Sphere Formation Assays

For sphere formation assays, the indicated cells (1 × 10^3^) were trypsinized and resuspended in stem cell medium consisting of DMEM/F-12 (Gibco, United States), 1 × B27 (Invitrogen), 20 ng/ml epidermal growth factor (Invitrogen), 20 ng/mL basic fibroblast growth factor (Invitrogen), and 2 mM L-glutamine (Invitrogen) and seeded into 24-well ultralow attachment plates. Then, the cells were cultured at 37°C, and the medium was refreshed every 48 h. Two weeks later, the stemness spheres were observed and counted under an optical microscope.

### Lentiviral Production and Cell Transfection

Lentiviral production and cell transfection were performed as we previously described (Liu et al., 2020b). Briefly, recombinant lentivirus for overexpression of HCG18 or UBE2O in BC cells and corresponding negative controls were designed and synthesized by GenePharma (Shanghai, China). The recombinant lentivirus was transfected into the BC cells with polybrene (8 μg/ml). Twenty-four hours after transfection, the cells were selected with puromycin, and qRT-PCR was applied to detect the transfection efficiency.

To establish stable HCG18- or UBE2O-reducing BC cells, shRNAs targeting HCG18 or UBE2O were designed by Sigma and transfected into the indicated cells according to the manufacturer’s instructions. The shRNA sequences are listed in Supplementary Table S3. qRT-PCR was used to detect the transfection efficiency.

Small interfering RNAs (siRNAs) targeting HIF-1α and control siRNAs were designed by GenePharma. The miRNA mimics and inhibitor were synthesized by Sigma. For transfection, the indicated cells were seeded into six-well plates (5 × 10^5^ per well) and cultured at 37°C overnight. Then, the cells were transfected with the corresponding vectors using Lipofectamine 2,000 transfection reagent (Invitrogen) according to the manufacturer’s instructions. Forty-eight hours later, the transfected cells were harvested for follow-up experiments. The siRNA sequences are listed in Supplementary Table S3.

### Subcellular Fractionation

Nuclear and cytoplasmic RNA separation was conducted using a PARIS™ Kit (Invitrogen) according to the manufacturer’s instructions. qRT-PCR was used to analyze the expression of HCG18 in different fragments of BC cells.

### RNA Immunoprecipitation Assay

RNA immunoprecipitation (RIP) assays were performed to validate the interaction of miR-103a-3p with HCG18, UBE2O, or AGO2 using the Magna RIP™ RNA-Binding Protein Immunoprecipitation Kit (Millipore, United States). To validate the interaction between miR-103a-3p and HCG18 or UBE2O, HEK293T cells were cotransfected with MS2bs vectors cloned with related DNA sequences (MS2bs, MS2bs-HCG18-WT, MS2bs-HCG18-Mut, MS2bs-UBE2O-3′UTR-WT, or MS2bs-UBE2O-3′UTR-Mut) and MS2bp-GFP overexpression vector (GenePharma, China). To validate the interaction between miR-103a-3p and AGO2, the indicated cells (MDA-MB-231^*NC/shHCG18*^ and MCF-7^*NC/HCG18*^) were transfected with miR-103a-3p mimics. Then, the cells were collected and lysed with RIPA. After that, the lysate was subjected to RIP with an anti-GFP or anti-AGO2 antibody (Abcam, United States) according to the manufacturer’s instructions. Finally, the samples were treated with proteinase K (Merck, United States), and qRT-PCR was used to analyze the binding of target RNA and protein.

### Dual-Luciferase Reporter Assay

To assess the regulatory effects of HCG18 on miR-103a-3p and miR-103a-3p on UBE2O mRNA, dual-luciferase reporter assays were performed using a double-luciferase assay system (Promega, United States). In brief, 3′ untranslated region (UTR) of UBE2O (wild/mutant type) or HCG18 (wild/mutant type) luciferase reporter plasmids and miR-103a-3p mimics were cotransfected into HEK-293T cells. Then, the cells were lysed, and luciferase assays were performed according to the manufacturer’s instructions. Firefly luciferase activity normalized to Renilla luciferase activity was used as an internal control.

To analyze whether HIF-1α could bind to the promoter region of HCG18, HEK-293T cells were cotransfected with luciferase reporter plasmids encoding the HCG18 promoter region (wild/mutant type) and with HIF-1α plasmids. Forty-eight hours later, the cells were harvested, and dual-luciferase assays were performed. Firefly luciferase activity was normalized to Renilla luciferase activity (used as an internal control).

### Chromatin Immunoprecipitation

Chromatin immunoprecipitation (ChIP) assays were performed as previously reported (Liang et al., 2020). Briefly, the indicated cells were treated with formaldehyde to crosslink the target protein and chromatin and sonicated to an average length of 200 to 500 bp on ice. Then, a DNA depuration kit (Beyotime) was used to extract and clean the DNA fragments. After that, ChIP assays were conducted with an anti–HIF-1α antibody (catalog no. 36169S; CST) or immunoglobulin G (IgG) control. qRT-PCR was used to detect the promoter fragments of HCG18. The primers used in our experiment are listed in the Supplementary Material.

### Animal Study

All animal studies were approved by the Medical Experimental Animal Care Commission of Harbin Medical University and performed in the Second Affiliated Hospital of Harbin Medical University Laboratory. Animals used in our study were raised in specific pathogen-free animal facilities (temperature was maintained at 25°C with a 12-h light–dark cycle) and provided free access to clean water and food. The animals were anaesthetized with 1–3% isoflurane and humanely sacrificed by CO_2_ inhalation as previously reported (Liu et al., 2020c). For the tumorigenesis assay, 6-week-old BALB/c nude mice (Vital River, Beijing, China) were randomly assigned into two groups (*n* = 6), and then MDA-MB-231^*NC*^ or MDA-MB-231^*HCG18-sh*^ (5 × 10^5^) was injected into the mammary fat pads of mice. Tumor growth was measured every week and 7 weeks after injection. The mice were humanely sacrificed, and the tumors were extracted and fixed in formalin solution. The tumor volumes were evaluated by the following formula: 1/2 [length × width (Wang et al., 2019)]. For the lung metastasis model, MDA-MB-231^*NC*^ or MDA-MB-231^*HCG18-sh*^ (5 × 10^5^) cells were injected into the tail vein of 6-week-old BALB/c nude mice. Seven weeks after injection, the mice were humanely sacrificed, and the lungs were collected to count metastatic nodules and for HE staining.

### Statistical Analysis

All the data are presented as the means ± SD from at least three independent experiments. A normality test was used to analyze the distribution of all datasets. Differences between two groups were analyzed by Student *t* tests, and differences among multiple groups were analyzed by one-way analysis of variance. The *χ* ^2^ test was used to analyze the relationship between HCG18 expression and the clinicopathological features of BC patients. The Kaplan–Meier method and log-rank test were applied to generate and assess survival curves. *p* < 0.05 indicated statistical significance, which was evaluated by GraphPad Prism 8.0 software.

## Results

### The Expression Level of HCG18 Was Commonly Upregulated in BC and Was Associated With a Poor Prognosis in BC Patients

**FIGURE 1:**
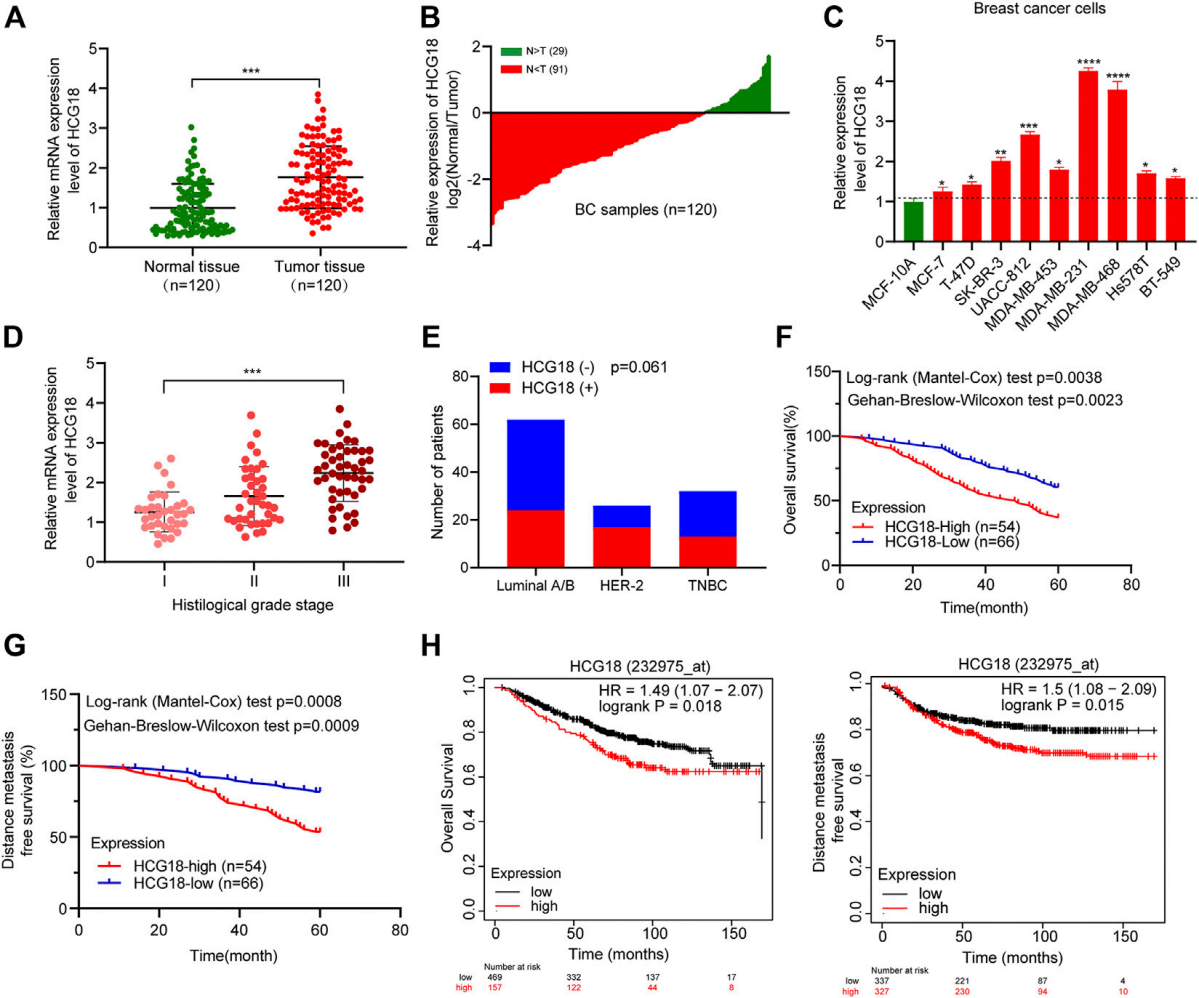
**(A**,**B)** qRT-PCR was used to detect the expression profile of HCG18 in BC tissues and normal mammary tissues. **(C)** HCG18 was detected by qRT-PCR in a panel of BC cell lines and a normal breast epithelial cell line (MCF-10A). **(D)** qRT-PCR was applied to measure HCG18 expression in BC patients with different histological grades. **(E)** The *χ* ^2^ test was applied to analyze the relationship between HCG18 expression and different subtypes of BC (triple-negative breast cancer [TNBC]). **(F,G)** Kaplan–Meier analysis showed that BC patients with high HCG18 expression had worse DMFS or OS than those with low HCG18 expression (the mean value was used to distinguish high or low HCG18 expression status). **(H)** Survival information of BC patients with high or low HCG18 expression was obtained from the Kaplan–Meier Plotter database. *p < 0.05, **p < 0.01, ***p < 0.001. The data represent at least three independent experiments.

A previous study reported that HCG18 was upregulated in BC tissues compared with normal tissues in the TCGA database (Xu et al., 2017). However, the biological function of HCG18 in BC is largely unknown. To investigate this, we first detected the HCG18 expression profile in BC tissues and corresponding normal breast tissues. The results showed that HCG18 was significantly upregulated in BC tissues compared with normal mammary tissues (Figures 1A,B). qRT-PCR was applied to detect HCG18 expression in a panel of BC cell lines and the mammary epithelial cell line MCF-10A. Figure 1C shows that MDA-MB-231 cells, which are highly invasive BC cells, had the highest HCG18 expression level, and HCG18 expression was lower in MCF-7 cells with low malignant potential. However, MCF-10A cells had the lowest HCG18 expression among all assessed BC cells. Then, we sought to assess the associations between HCG18 expression and clinicopathologic features in BC patients. We divided the samples into a HCG18 high-expression group and HCG18 low-expression group according to the average value of HCG18 expression in BC tissues. The results in Figures 1D,E and Table 1 show that HCG18 was positively correlated with the histological and clinical stage, tumor size, and axillary lymph node metastasis in BC patients. However, there were no differences in HCG18 expression in different subtypes of BC (Figure 1E), which was further confirmed by data from the lnCAR database (Supplementary Figures S1A–C). Figures 1F,G show that BC patients with high HCG18 expression had poor distant metastasis-free survival (DMFS) and overall survival (OS), which was also supported by survival information in the Kaplan–Meier Plotter database (Figure 1H). Collectively, these results revealed that HCG18 was commonly overexpressed in BC patients and that patients with high HCG18 expression tended to have a higher risk of metastasis and a poorer prognosis than those with low expression.

**TABLE 1:** Association HCG18 expression and patients’ clinicopathological characteristics in invasive ductal carcinoma tissues.

Characteristics	HCG18 expression		No	*p* Value
	High	Low		
Age			120	ns
≤40	5	5		
>40	49	61		
ER/PR			120	ns
(+)	24	38		
(−)	30	28		
HER-2			120	ns
0–2 (+)	36	45		
3 (+)	18	21		
Ki-67			120	0.0431
≤20%	22	40		
>20%	32	26		
Tumor size			120	0.0259
T1	17	35		
T2–T3	37	31		
Axillary lymph node metastasis			120	0.0002
N0	22	50		
N1–N3	32	16		
Histological grade				
1–2 grade	38	54	120	ns
3 grade	16	12		
Clinical stages			120	<0.0001
І–IIA	19	55		
IIB–III	35	11		
Statistically significant difference (*p* < 0.05) was indicated in bold letters.				

### Aberrant Expression of HCG18 Was Associated With the Proliferation, Metastasis, and CSPs of BC Cells

**FIGURE 2:**
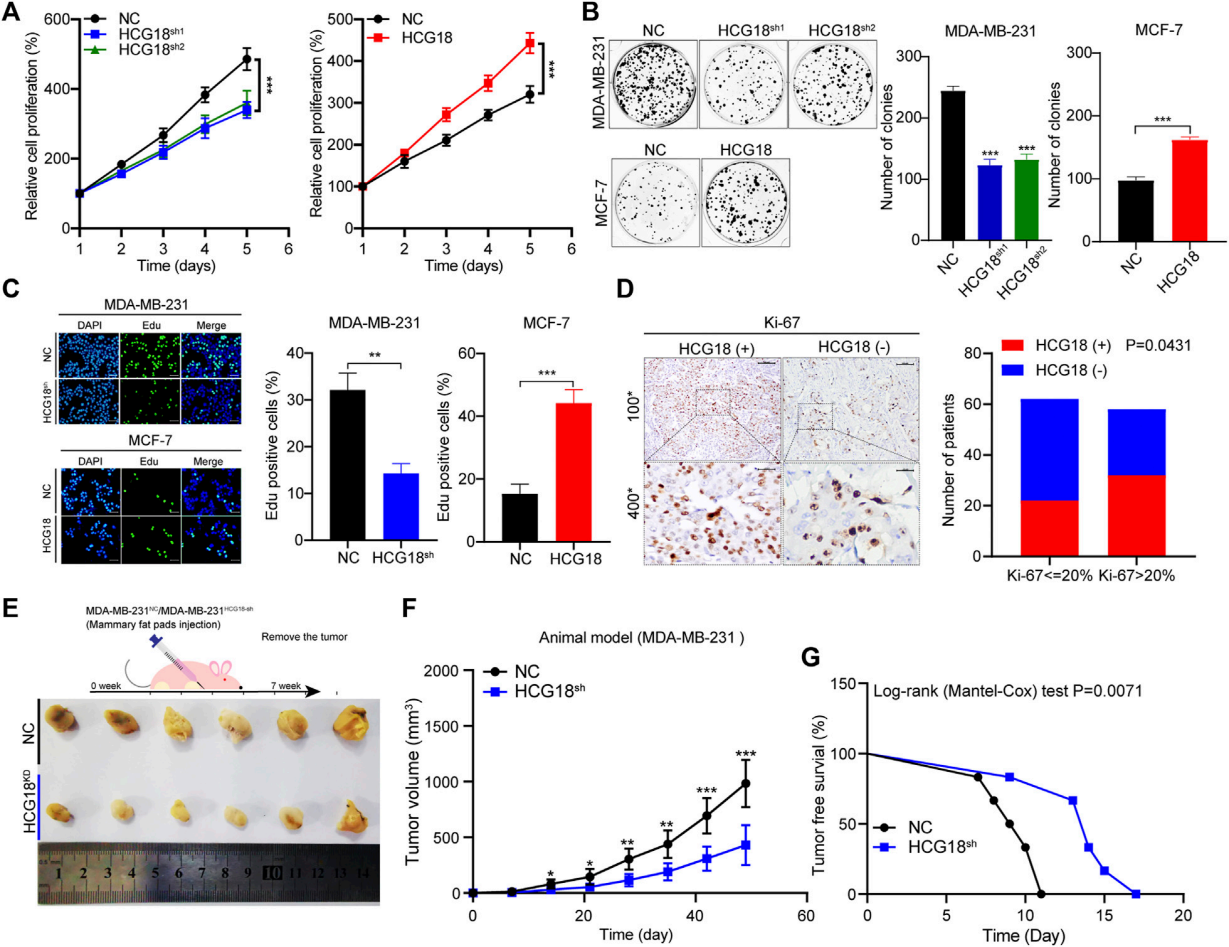
**(A**,**B)** CCK-8 **(A)** and colony formation assays **(B)** were performed to detect the proliferation ability of MDA-MB-231^*NC*^/MDA-MB-231^*HCG18-sh*^ and MCF-7^*NC*^/MCF-7^*HCG18*^ cells. **(C)** EdU staining assays showed that HCG18 promoted BC cell proliferation *in vitro* (scale bar, 50 μm). **(D)** The expression level of Ki-67 in BC patients was detected by IHC (upper: magnification ×100; scale bar, 100 μm; lower: magnification ×400; scale bar, 20 μm), and the correlation between HCG18 and Ki-67 was analyzed by a *χ* ^2^ test (Ki-67 <20% was regarded as low expression). **(E–G)** Mouse xenograft models were used to investigate the tumorigenesis of MDA-MB-231^*NC*^/MDA-MB-231^*HCG18-sh*^ cells *in vivo* **(E)**; the volumes of tumors were recorded **(F)**, and the tumor-free survival of the two groups was analyzed **(G)**. The data are shown as the mean ± SD. *p < 0.05, **p < 0.01, ***p < 0.001. The data represent at least three independent experiments.

To investigate the biological roles of HCG18 in BC, we used MDA-MB-231, which has high HCG18 expression, and MCF-7 cells, which have low HCG18 expression, for further experiments. Three sets of specific shRNAs targeting HCG18 were transfected into MDA-MB-231 cells to generate HCG18 reducing cells (MDA-MB-231^*HCG18-sh1*^, MDA-MB-231^*HCG18-sh2*^, and MDA-MB-231^*HCG18-sh3*^), and HCG18 was stably overexpressed in MCF-7 cells (MCF-7^*HCG18*^). The HCG18 expression profile in these newly generated cells was detected by qRT-PCR. Supplementary Figure S1D shows that HCG18-sh1 and HCG18-sh2 yielded a better reducing efficiency than HCG18-sh3 in MDA-MB-231 cells, and HCG18 was significantly upregulated in MCF-7^*HCG18*^ cells, so MDA-MB-231^*HCG18-sh1*^, MDA-MB-231^*HCG18-sh2*^, and MCF-7^*HCG18*^ cells were used in the following experiments. The CCK-8 assay is a viability assay that provides a readout, that is, proportional to cell number; this approach is often applied to detect the proliferative capacity of cancer cells. We used CCK-8 assays to evaluate the effect of HCG18 alterations on proliferation. Figure 2A shows that the proliferation capability was significantly decreased after reducing HCG18 in MDA-MB-231 cells, whereas MCF-7^*HCG18*^ cells exhibited a better proliferation ability than the control cells. Colony formation assays (Figure 2B) and EdU staining assays (Figure 2C) further confirmed this conclusion. Then, the expression of Ki-67 (the nuclear expression of Ki-67 can be evaluated to assess tumor proliferation capability; Ki-67 >20% was regarded as high Ki-67 expression profile) was detected in BC tissues by IHC, and the *χ* ^2^ test was used to analyze the relationship between HCG18 and Ki-67 expression. Figure 2D shows the positive correlation between HCG18 and Ki-67 expression in clinical samples of BC patients. To verify the effect of HCG18 on tumorigenesis *in vivo*, MDA-MB-231^*NC*^ and MDA-MB-231^*HCG18-sh1*^ cells were orthotopically injected into the mammary fat pads of BALB/c nude mice. As shown in Figure 2E, reducing HCG18 significantly suppressed tumor growth and prolonged tumor-free survival compared with that in the control group. Taken together, these results demonstrated that HCG18 could promote BC cell proliferation both *in vitro* and *in vivo*.

**FIGURE 3:**
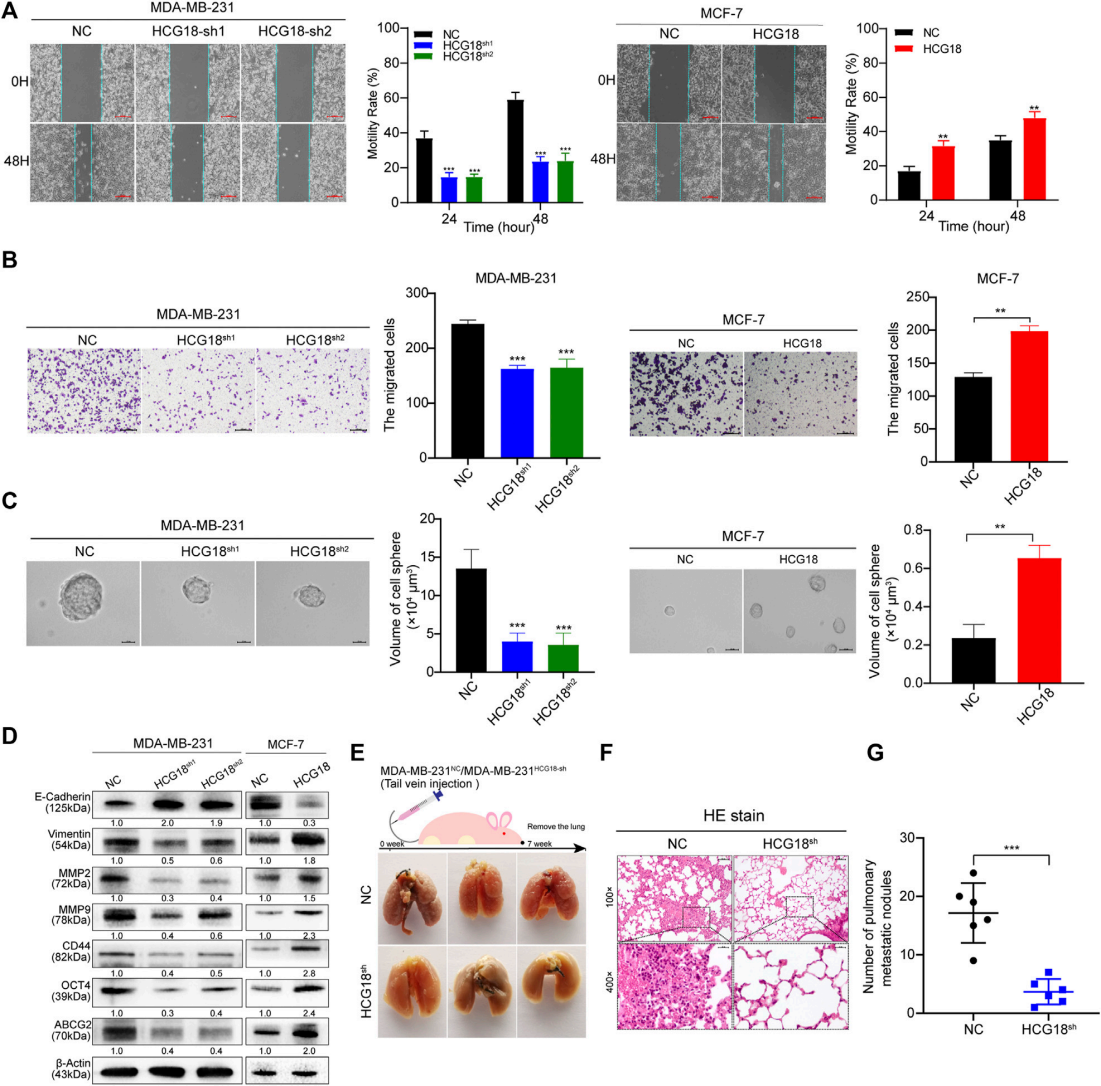
**(A)** Wound healing assays were performed to detect the effect of HCG18 on the migration ability of BC cells (scale bar, 200 μm). **(B)** The invasion capability of MDA-MB-231^*NC*^/MDA-MB-231^*HCG18-sh*^ and MCF-7^*NC*^/MCF-7^*HCG18*^ cells was assessed by Matrigel invasion assays (scale bar, 200 μm). **(C)** The spheroid formation capabilities of BC cells after changing HCG18 expression were examined by tumor sphere formation assays (scale bar, 20 μm). **(D)** The expression levels of EMT markers (E-cadherin and vimentin), metastatic markers (MMP-2 and MMP-9), and CSP markers (CD44, OCT4, and ABCG2) were determined by Western blotting assays. **(E–G)** Lungs excised from mice in the MDA-MB-231^*NC*^/MDA-MB-231^*HCG18-sh*^ lung metastatic models were photographed **(E)** and subjected to HE staining **(F)**, and then the lung metastasis nodes were analyzed **(G)**. The data are shown as the mean ± SD. *p < 0.05, **p < 0.01, ***p < 0.001. The data represent at least three independent experiments.

The results in Table 1 show that there was a close relationship between HCG18 expression and the status of axillary lymph node metastasis in BC patients, so we focused on the prometastatic function of HCG18 in BC. Wound healing assays showed that the migration ability was extremely decreased after reducing HCG18 in MDA-MB-231 cells, and the migration ability of MCF-7^*HCG18*^ cells was increased in comparison with that of the control group (Figure 3A). Matrigel invasion assays also showed similar results (Figure 3B). Western blotting assays revealed that the epithelial-related protein E-cadherin was upregulated, but the mesenchymal marker vimentin and the metastatic markers MMP-2 and MMP-9 were downregulated in MDA-MB-231^*HCG18-sh*^ cells compared with control cells. The opposite results were observed in MCF-7^*HCG18*^ cells (Figure 3D and Supplementary Figure S1E).

In recent years, a growing number of studies have reported that CSPs play important roles in tumor survival, proliferation, invasion, immune escape, and drug resistance. Thus, the following experiments explored the relationship between HCG18 expression and CSPs in BC cells. Sphere formation assays revealed that the sphere formation ability of MDA-MB-231^*HCG18-sh*^ cells was obviously decreased compared with that of the control cells; however, the CSPs were remarkably enhanced after overexpressing HCG18 in MCF-7 cells (Figure 3C). Western blotting assays showed that the CSP markers CD44, ABCG2, and OCT4 were markedly downregulated in MDA-MB-231^*HCG18-sh*^ cells and increased in MCF-7^*HCG18*^ cells compared with the control cells (Figure 3D and Supplementary Figure S1E). Finally, lung metastasis mouse models were used to investigate the prometastatic function of HCG18 *in vivo*. The results in Figures 3E–G show that mice injected with MDA-MB-231^*HCG18-sh*^ cells exhibited fewer metastatic nodes than the mice injected with control cells, which indicates that HCG18 could promote BC cell lung metastasis *in vivo*. Collectively, these results confirmed that HCG18 could endow BC cells with CSPs *in vitro* and promote BC cell metastasis both *in vitro* and *in vivo*.

### UBE2O Contributed to the Tumor-Promoting Role of HCG18 in BC Cells

**FIGURE 4:**
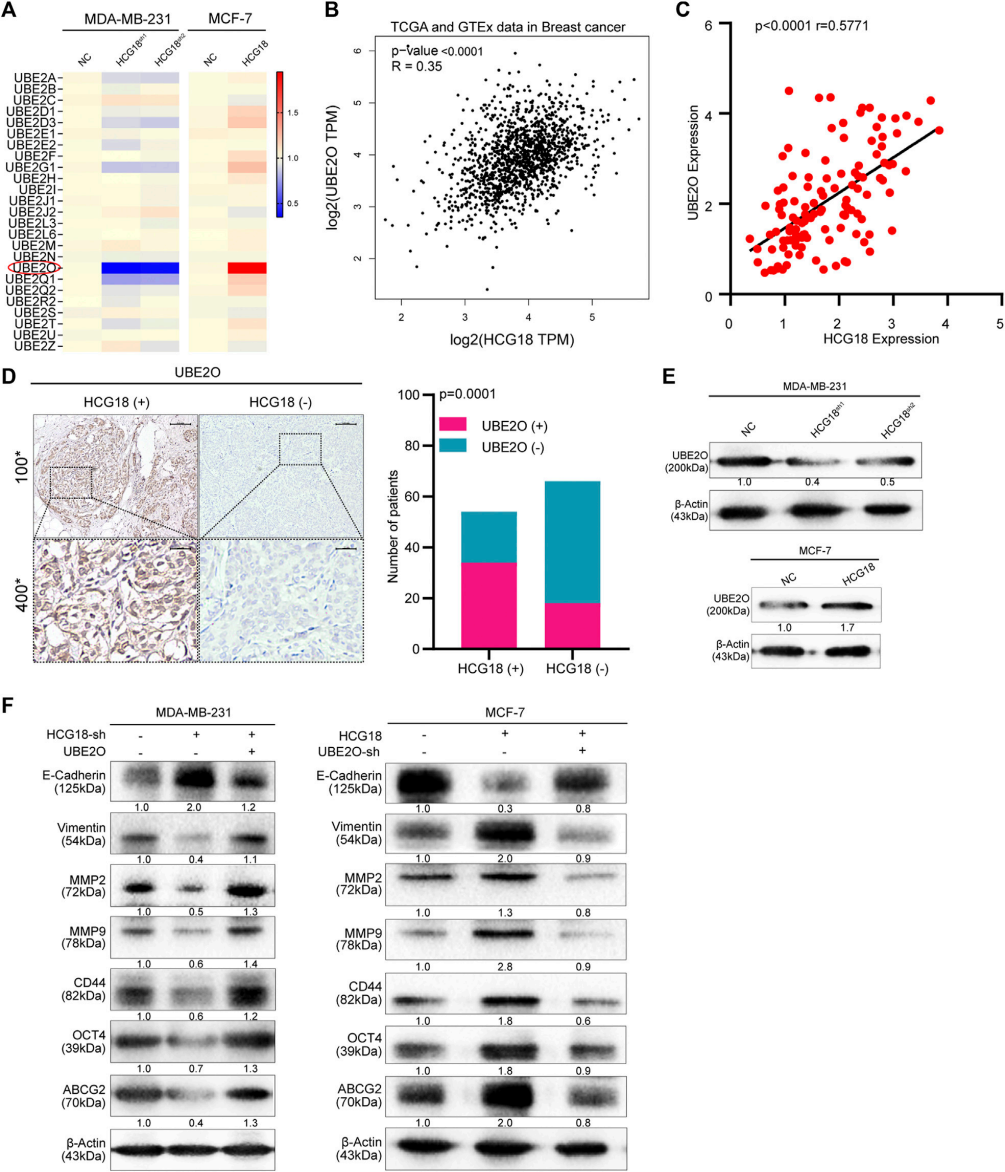
**(A)** The expression levels of the predicted tumor-related E2s targeting HCG18 were analyzed in MDA-MB-231^*NC*^/MDA-MB-231^*HCG18-sh*^ and MCF-7^*NC*^/MCF-7^*HCG18*^ cells. **(B)** Result from the GEPIA database indicated that UBE2O expression was positively correlated with HCG18 expression in BC. **(C)** The expression level of UBE2O in BC patients was detected by qRT-PCR, and Pearson correlation analysis was used to detect the relationship between UBE2O and HCG18 in BC patients. **(D)** The protein expression level of UBE2O was detected by IHC in BC tissues (the staining results were evaluated with the H-SCORE system, and the mean value was used to distinguish high or low UBE2O expression status; upper: magnification ×100; scale bar, 100 μm; lower: magnification ×400; scale bar, 20 μm); its correlation with HCG18 was analyzed by the *χ* ^2^ test. **(E)** Western blotting assays were used to detect UBE2O expression after changing HCG18 expression in the indicated cells. **(F)** We overexpressed UBE2O in MDA-MB-231^*HCG18-sh*^ cells (generating MDA-MB-231^*HCG18-sh+UBE2O*^ cells) and reducing UBE2O in MCF-7^*HCG18*^ cells (generating MCF-7^*HCG18+UBE2O−sh*^ cells). Then, Western blotting assays were performed to examine the changes in the expression of EMT markers (E-cadherin and vimentin), metastatic markers (MMP-2 and MMP-9), and CSP markers (CD44, OCT4, and ABCG2) in the indicated cells. The data are shown as the mean ± SD. *p < 0.05, **p < 0.01, ***p < 0.001. The data represent at least three independent experiments.

E2s play important roles in regulating cellular posttranslational protein modifications and are involved in a wide range of key physiological or pathologic processes. Multiple studies have reported that aberrant E2 expression is commonly observed in a variety of human cancers. Some cancer-related E2s could facilitate DNA repair and cell cycle progression and activate oncogenic signaling pathways, which promote cancer progression and metastasis (Voutsadakis, 2013). Our previous study reported that UBE2O could promote proliferation and EMT and endow BC cells with CSPs by degrading AMPKα2, thus activating the mTORC1-MYC axis (Liu et al., 2020b). For this reason, we hypothesized that there may be a potential interaction network between HCG18 and E2s in BC. Then, a panel of cancer-related E2s was detected in MDA-MB-231^NC^/MDA-MB-231^*HCG18-sh*^ and MCF-7^*NC*^/MCF-7^*HCG18*^ cells. Coincidentally, we found that there was a close positive correlation between HCG18 and UBE2O expression in the indicated cells (Figure 4A and Supplementary Figure S1F). Correlation analysis with the Gene Expression Profiling Interactive Analysis (GEPIA) database showed that there was a positive correlation between HCG18 and UBE2O expression in BC (Figure 4B), which was further verified in clinical samples (Figures 4C,D). Next, we reduced UBE2O in MCF-7^*HCG18*^ cells (generating MCF-7^*HCG18+UBE2O−sh*^ cells) and overexpressed UBE2O in MDA-MB-231^*HCG18-sh1*^ cells (generating MDA-MB-231^*HCG18-sh+UBE2O*^ cells). Western blotting assays showed that UBE2O was remarkably decreased in MDA-MB-231^*HCG18-sh*^ cells and that the expression level of UBE2O was increased in MCF-7^*HCG18*^ cells (Figure 4E and Supplementary Figure S2A). The results in Figure 4F and Supplementary Figure S2B revealed that the upregulation of E-cadherin and the downregulation of vimentin, MMP-2, MMP-9, CD44, and OCT4 were attenuated in MDA-MB-231^*HCG18-sh+UBE2O*^ cells, and the opposite results were observed in MCF-7^*HCG18+UBE2O−sh*^ cells compared with control cells.

**FIGURE 5:**
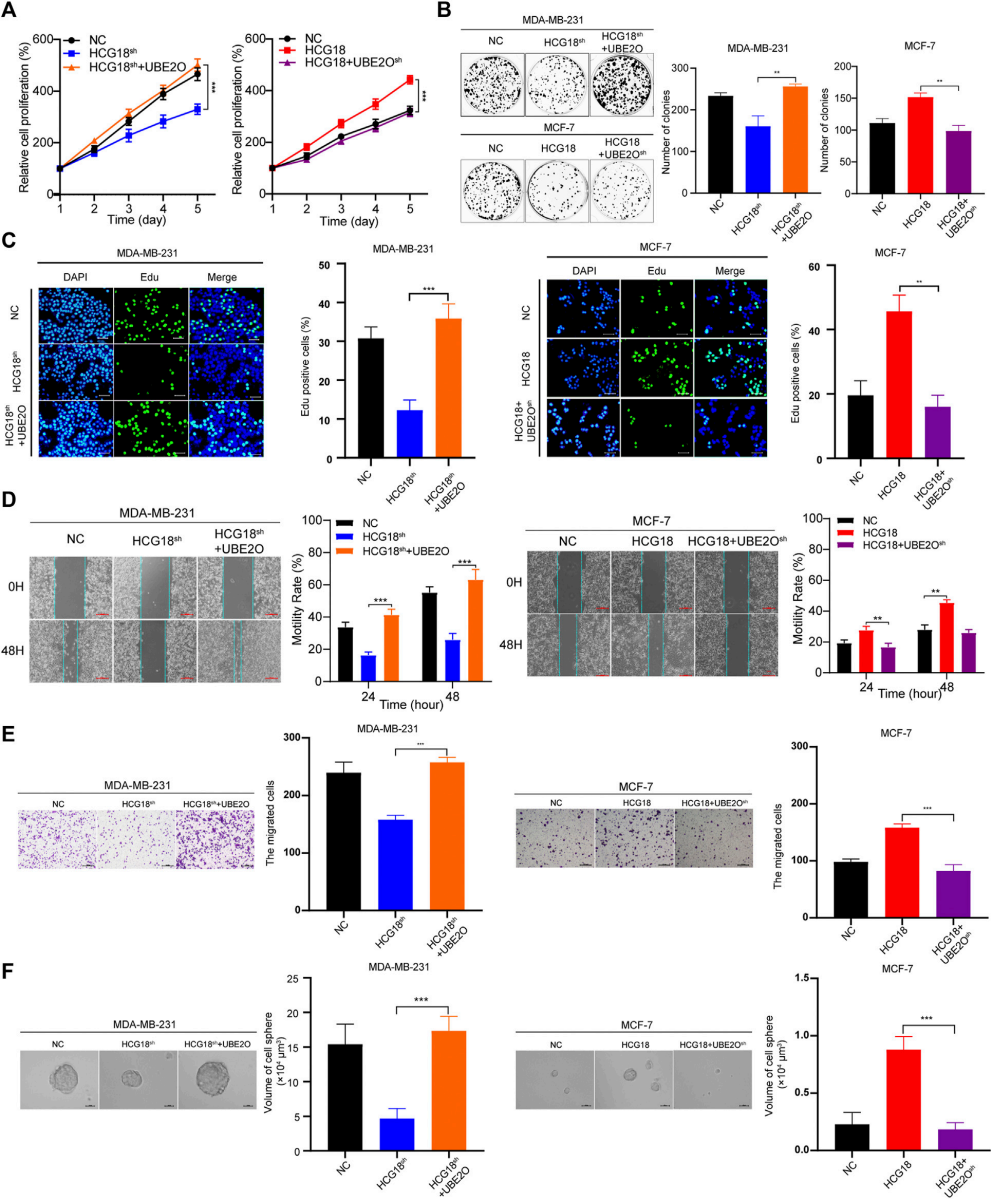
**(A–C)** CCK-8 **(A)**, colony formation **(B)** and EdU staining assays (scale bar, 50 μm) **(C)** were performed to detect the proliferation capabilities of MDA-MB-231^*NC*^/MDA-MB-231^*HCG18-sh*^/MDA-MB-231^*HCG18-sh+UBE2O*^ and MCF-7^*NC*^/MCF-7^*HCG18*^/MCF-7^*HCG18+UBE2O−sh*^ cells. **(D–F)** UBE2O was changed in MDA-MB-231 and MCF-7 cells, and scratch assays (scale bar, 200 μm) **(D)**, invasion assays (scale bar, 200 μm) **(E)**, and sphere formation assays (scale bar, 20 μm) **(F)** were performed to detect the migration, invasion, and sphere formation capabilities in the indicated cells. The data are shown as the mean ± SD. *p < 0.05, **p < 0.01, ***p < 0.001. The data represent at least three independent experiments.

Then, we explored the effect of UBE2O on the protumor function of HCG18 in BC cells *in vitro*. CCK-8 (Figure 5A), colony formation assays (Figure 5B), and EdU staining assays (Figure 5C) showed that overexpressing UBE2O could significantly reverse the decline in the proliferation ability of MDA-MB-231^*HCG18-sh*^ cells, and the enhancement of proliferative capacity was attenuated after reducing UBE2O in MCF-7^*HCG18*^ cells. Figures 5D–F show that the migration, invasion, and sphere formation capabilities of MDA-MB-231^*HCG18-sh+UBE2O*^ cells were improved compared with those of MDA-MB-231^*HCG18-sh*^ cells, and reducing UBE2O in MCF-7^*HCG18*^ cells remarkably destroyed these capabilities in comparison with those of the control cells. Taken together, these results confirmed that UBE2O plays a vital role in the cancer-promoting function of HCG18 in BC cells.

### HCG18 Promoted the Expression of UBE2O by Sponging miR-103a-3p in BC Cells

**FIGURE 6:**
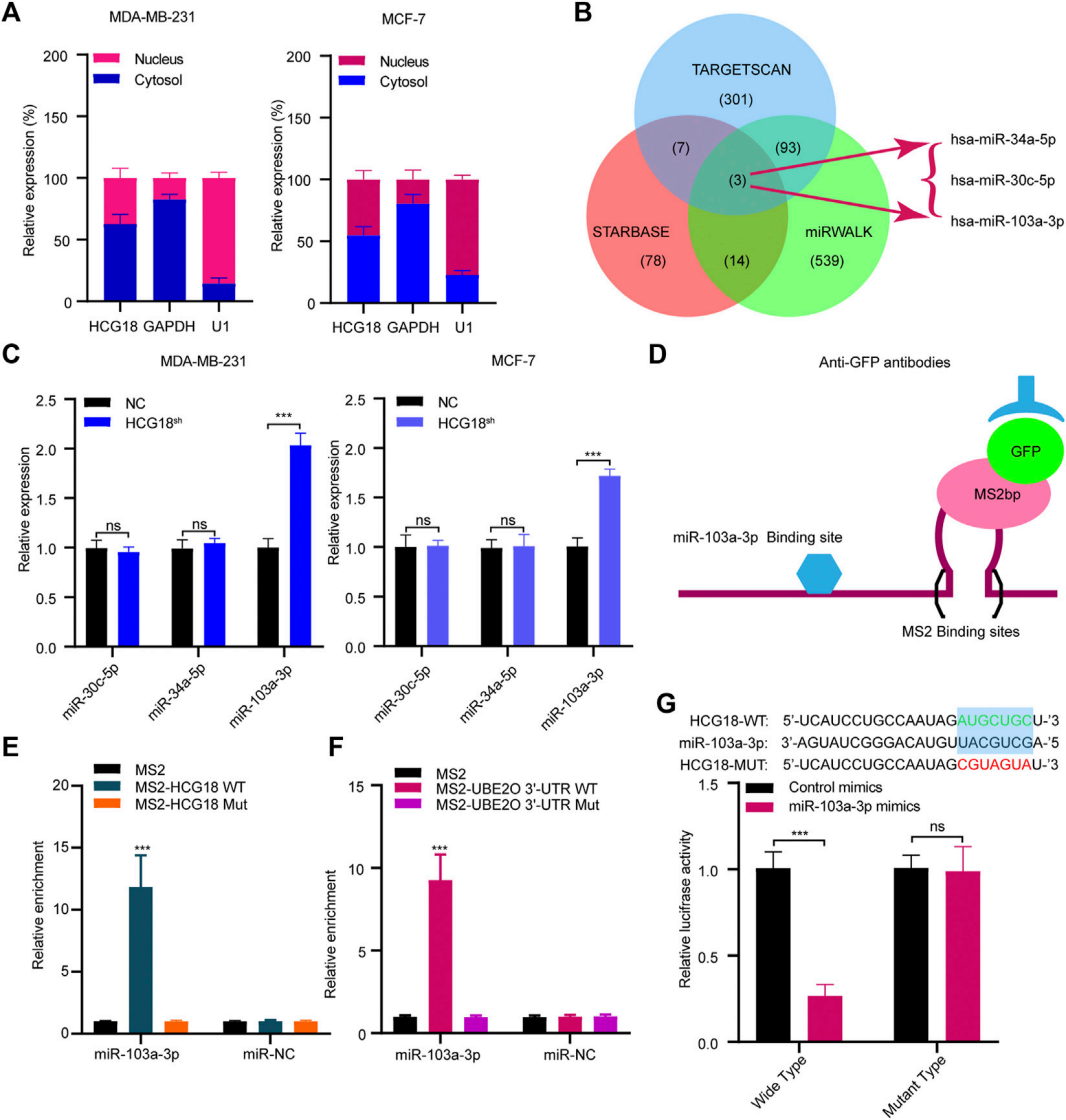
**(A)** Nucleocytoplasmic separation assays were used to confirm the cytoplasmic and nuclear distribution of components in BC cells. **(B)** Schematic illustration showing the overlapping miRNAs targeting both HCG18 and UBE2O using the TargetScan, miRWalk, and starBase databases. **(C)** qRT-PCR was used to determine the expression of predicted miRNAs in MDA-MB-231^*NC*^/MDA-MB-231^*HCG18-sh*^ and MCF-7^*NC*^/MCF-7^*HCG18-sh*^ cells. **(D,E)** MS2-RIP followed by miRNA qRT-PCR to detect the interactions between miRNAs and HCG18. **(F)** MS2-RIP followed by miRNA qRT-PCR to detect the interactions between miRNAs and UBE2O mRNA 3′-UTR. **(G)** HCG18-Wt/HCG18-mut and miR-103a-3p mimics were cotransfected into HEK-293T cells, and then dual-luciferase reporter assays were performed. The data are shown as the mean ± SD. *p < 0.05, **p < 0.01, ***p < 0.001. The data represent at least three independent experiments.

**FIGURE 7:**
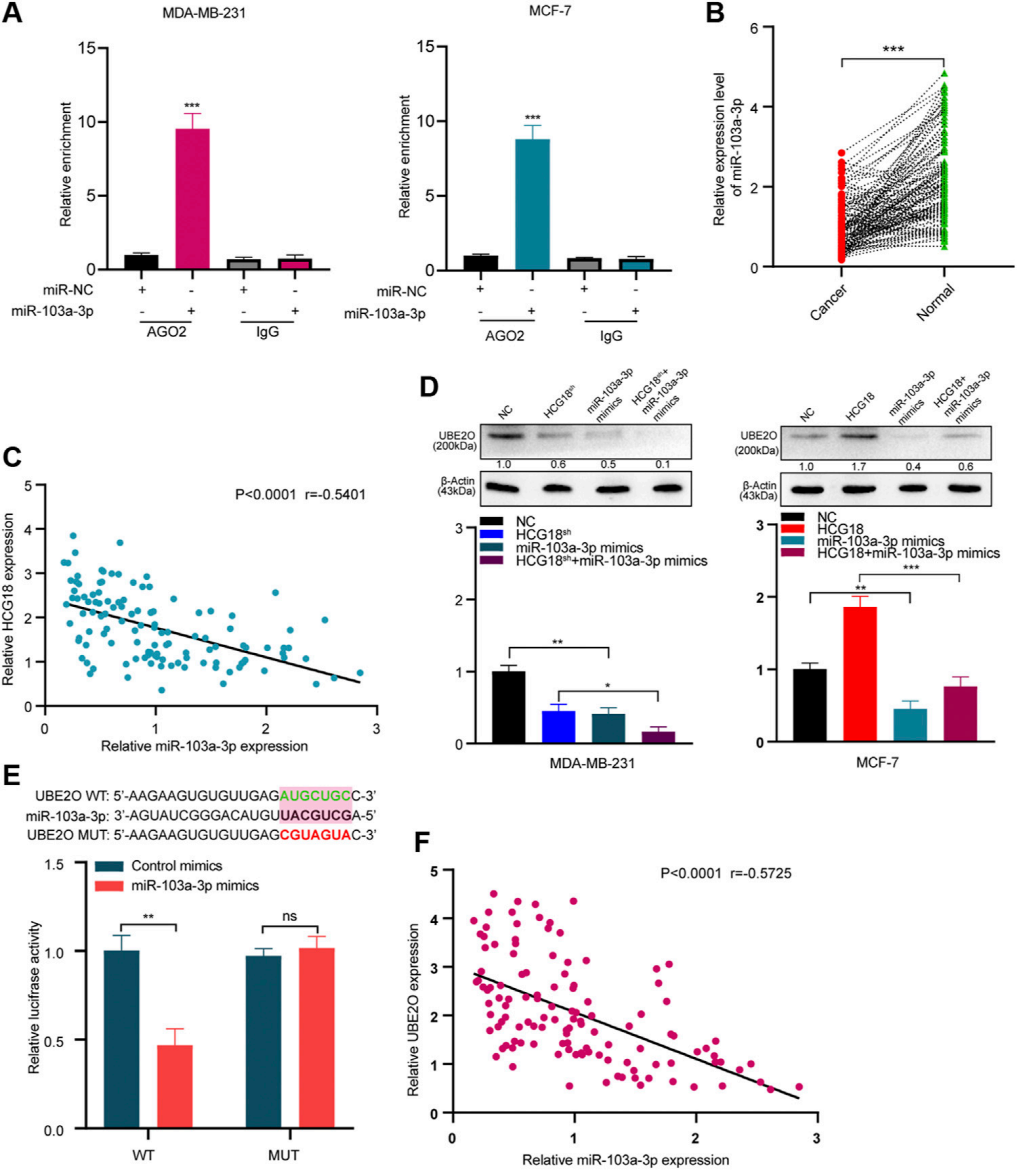
**(A)** Anti-AGO2 RIP assays were performed in BC cells transfected with miR-103a-3p mimics or NC, followed by qRT-PCR to detect the abundance of HCG18. Percent input method was used to normalize RIP-qPCR data. **(B,C)** The expression level of miR-103a-3p in BC tissues and normal tissues was measured by qRT-PCR **(B)**, and their correlation was determined by Pearson correlation analysis **(C)**. **(D)** miR-103a-3p mimics were transfected into MDA-MB-231^*NC*^/MDA-MB-231^*HCG18-sh*^ and MCF-7^*NC*^/MCF-7^*HCG18*^ cells, and then qRT-PCR and Western blotting assays were performed to detect the changes in UBE2O expression in the indicated cells. **(E)** UBE2O-WT/UBE2O-Mut and miR-103a-3p mimics were cotransfected into HEK-293T cells, and then dual-luciferase reporter assays were performed. **(F)** Pearson correlation coefficient was employed to analyze the relationship between miR-103a-3p and UBE2O in BC patients. The data are shown as the mean ± SD. *p < 0.05, **p < 0.01, ***p < 0.001. The data represent at least three independent experiments.

To further clarify the mechanism by which HCG18 regulates UBE2O in BC cells, we first ascertained the cellular location of HCG18 in BC cells. Subcellular fractionation assays showed that HCG18 was predominantly distributed in the cytoplasm of BC cells (Figure 6A), which was further confirmed by the lncLocator database (Supplementary Figure S2C) and was in accordance with previous studies (Xu et al., 2019; Li et al., 2020a). Recently, many studies confirmed that cytoplasmic lncRNAs could affect the biological process of BC cells by modulating the expression of miRNAs in a ceRNA-dependent manner (Liang et al., 2020; Ni et al., 2020). Therefore, we speculated that HCG18 could influence the stability of UBE2O mRNA *via* a ceRNA-mediated mechanism. To validate this, TargetScan, miRwalk, and starBase were used to predict the potential target miRNAs, and the results in Figure 6B revealed that there were three miRNAs (miR-30a-5p, miR-34a-5p, and miR-103a-3p) connected with both HCG18 and UBE2O. Then, we reduced HCG18 in MDA-MB-231 and MCF-7 cells, and qRT-PCR was applied to detect the changes in the expression of the three miRNAs above. The results showed that compared with that in the control group, the expression level of miR-103a-3p was remarkably increased after reducing HCG18 in both MDA-MB-231 and MCF-7 cells. However, there were no changes in the expression of the other two miRNAs in the indicated cells (Figure 6C). RIP assays showed that miR-103a-3p could associate with HCG18 *via* complementary binding sites, whereas the nontargeting miR-NC did not associate with HCG18 (Figures 6D,E). RIP assays also confirmed that miR-103a-3p could specifically bind to the UBE2O 3′UTR (Figure 6F). Dual-luciferase assays revealed that transfection of miR-103a-3p mimics could significantly reduce the luciferase activity of the HCG18-WT group but failed to affect that of the mutant group (Figure 6G). miRNAs degrade targeted mRNA in an Ago2-dependent manner by binding their targets. Next, we performed RIP in MDA-MB-231 and MCF-7 cells transiently overexpressing miR-103a-3p to pull down HCG18 using anti-Ago2 antibodies or control IgG, and qRT-PCR was used to analyze the enrichment of HCG18 in immunoprecipitates. The amount of HCG18 pulled down with anti-Ago2 antibodies was remarkably increased in cells transfected with miR-103a-3p mimics compared with control cells (Figure 7A). Then, the expression level of miR-103a-3p was detected in BC tissues and corresponding normal tissues. The results showed that miR-103a-3p was obviously downregulated in BC tissues compared with normal breast tissues (Figure 7B). Finally, we found that there was a negative relationship between miR-103a-3p and HCG18 in BC tissues (Figure 7C). Collectively, these results revealed that HCG18 acts as a sponge for miR-103a-3p in BC cells.

Then, we validated whether miR-103a-3p could mediate the function of HCG18 in promoting UBE2O expression. The results in Figure 7D and Supplementary Figure S2D show that ectopic expression of miR-103a-3p by miRNA mimics obviously reduced UBE2O expression in the indicated cells. Dual-luciferase assays further revealed that overexpression of miR-103a-3p could decrease the luciferase activity of the wild-type UBE2O reporter but not the mutant UBE2O reporter, which indicated that UBE2O was a direct target of miR-103a-3p (Figure 7E). The correlation analysis in Figure 7F identified that UBE2O expression was negatively correlated with miR-103a-3p expression in BC tissues. Finally, we detected the biological functions of miR-103a-3p in BC cells. EdU staining assays (Supplementary Figure S2E), invasion assays (Supplementary Figure S2F), and scratch assays (Supplementary Figure S2G) demonstrated that miR-103a-3p could significantly abolish the proliferation, migration, and invasion capabilities of MCF-7 cells after upregulating HCG18 in MCF-7 cells. The results in Supplementary Figure S2H revealed that the decreased expression of E-cadherin and increased expression of vimentin, MMP-2, MMP-9, CD44, and OCT4 were remarkably reversed by miR-103a-3p mimics. Taken together, these results claimed that HCG18 promoted UBE2O expression by sponging miR-103a-3p in human BC.

### HIF-1α Transcriptionally Promoted HCG18 Expression in BC Cells

**FIGURE 8:**
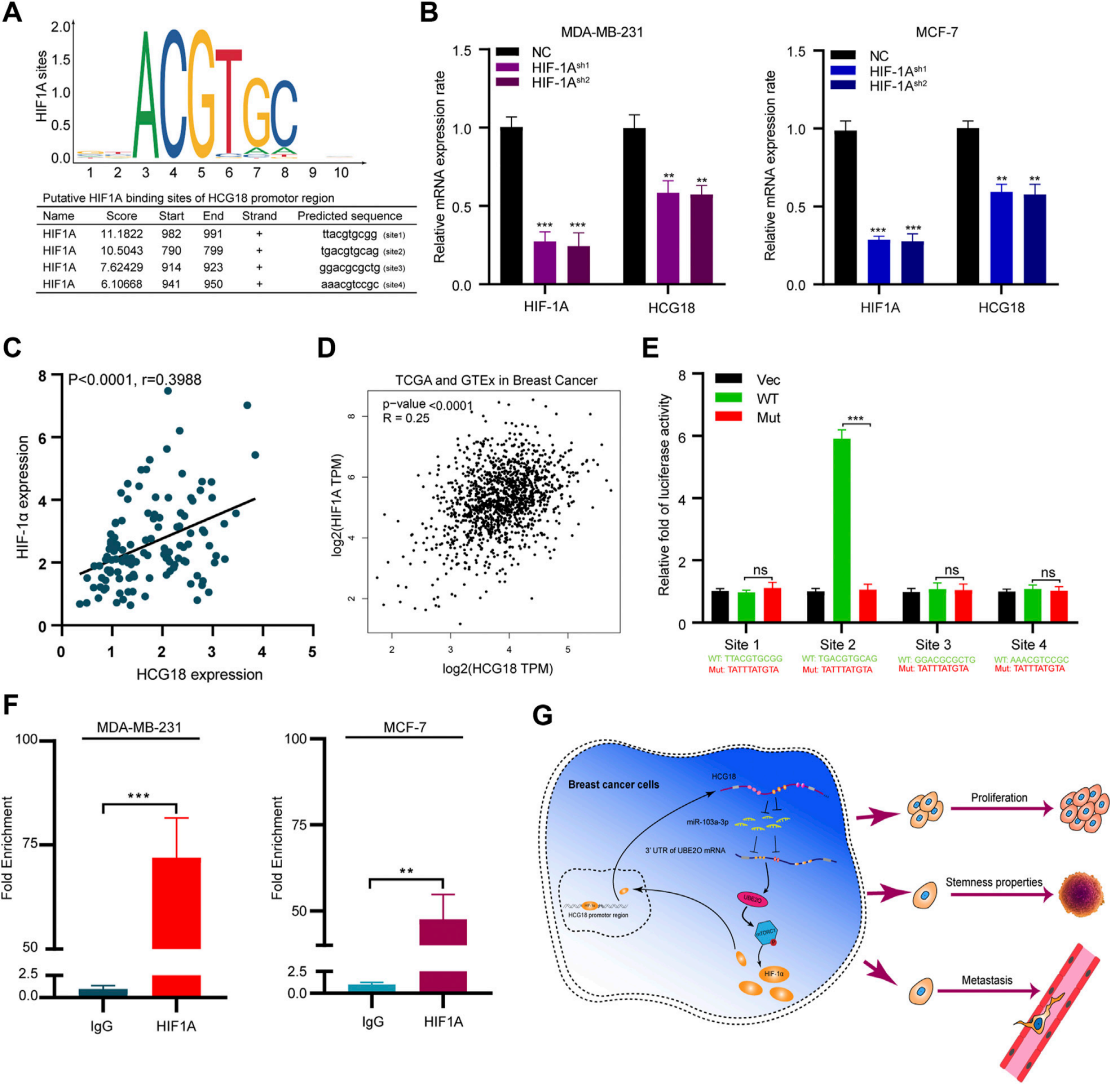
**(A)** HIF-1α was predicted as a potential transcription factor of HCG18 by the JASPAR database, and the putative binding sites are listed. **(B)** HIF-1α was reduced in MDA-MB-231 and MCF-7 cells, and qRT-PCR was applied to determine HIF-1α and HCG18 expression in the indicated cells. **(C)** HIF-1α was detected by qRT-PCR in BC tissues, and its correlation with HCG18 was analyzed by Pearson correlation coefficient analysis. **(D)** Result from GEPIA showed that there was a positive correlation between HIF-1α and HCG18 in BC. **(E)** Wild-type or mutant HCG18 promoter reporter constructs targeting the four binding sites of HIF-1α were separately transfected into 293T cells, HIF-1α plasmids were also transfected, and dual-luciferase reporter assays were performed. **(F)** ChIP assays were performed with IgG or HIF-1α antibodies in MDA-MB-231 and MCF-7 cells. Fragments of the HCG18 promoter region were detected by qRT-PCR. The data are shown as the mean ± SD. **(G)** Schematic diagram of the oncogenic role of HCG18 in BC cells. The HCG18/miR-103a-3p/UBE2O/HIF-1α axis constitutes a positive feedback loop in BC cells that plays an oncogenic role in BC pathogenesis. *p < 0.05, **p < 0.01, ***p < 0.001. The data represent at least three independent experiments.

As we verified that HCG18 was upregulated in BC, subsequent experiments were carried out to explore the regulatory mechanism resulting in the aberrant expression of HCG18 in BC. The JASPAR database was applied to identify the potential HCG18 transcription factors, and coincidentally, we found that there were four potential binding sites for HIF-1α in the promoter region of HCG18 (Figure 8A). Our previous study demonstrated that UBE2O could activate the mTORC1 signaling pathway by meditating AMPKα2 ubiquitination and that mTORC1 could regulate HIF-1α expression, which in turn promoted cell growth and anabolism (Laplante and Sabatini, 2012; Vila et al., 2017). Therefore, HIF-1α was reduced in MDA-MB-231 and MCF-7 cells. The efficiency of HIF-1α silencing and the relative HCG18 expression were detected by qRT-PCR. The results in Figure 8B revealed that HCG18 expression was significantly decreased after reducing HIF-1α in the indicated cells. Correlation analysis showed that there was a positive relationship between HIF-1α and HCG18 expression in BC tissues (Figure 8C), which was further confirmed with the GEPIA database (Figure 8D). Then, four sets of luciferase reporter plasmids including the wild-type or mutant HCG18 promoter region were constructed, and dual-luciferase assays were performed to validate the potential HIF-1α binding sites. For site 2, the transfection of HIF-1α plasmids could significantly increase the luciferase activity of the WT group but not the mutant group. For the other three sites, however, there were no differences in luciferase activity between the WT and mutant groups after HIF-1α plasmid transfection (Figure 8E). These results indicated that HIF-1α could bind to the promoter region of HCG18 (site 2) and facilitate HCG18 transcription. This conclusion was further confirmed by ChIP assays (Figure 8F). Taken together, these results demonstrate that HIF-1α could promote HCG18 expression, thus forming a positive feedback loop in BC cells (Figure 8G).

## Discussion

From the results above, we can draw the following conclusions: 1) HCG18 is upregulated in BC tissues, and patients with high HCG18 expression tend to have a poor prognosis. 2) HCG18 can promote BC cell proliferation, invasion, and CSPs *in vitro* and facilitate breast tumor growth and metastasis *in vivo*. 3) HCG18 promotes the malignant phenotype of BC cells by sponging miR-103a-3p, indirectly enhancing UBE2O expression and activating the mTORC1 signaling pathway. 4) As a downstream target of the mTORC1 axis, HIF-1α can transcriptionally promote HCG18 expression and form a positive feedback loop in BC.

A growing number of studies have illuminated that the dysregulation of lncRNAs is involved in diverse pathologic processes, including cancer initiation and progression. The biological mechanism of lncRNAs involved in cancers can be summarized as follows: 1) cytoplasmic lncRNAs can competitively adsorb miRNAs (acting as ceRNAs), reducing the loss of function of miRNAs in controlling target genes. 2) LncRNAs can influence certain cell activities by combining with some epigenetic-related proteins and changing their subcellular localization, which may modify specific protein translation after transcription. 3) LncRNAs can control gene expression by associating with chromosomal DNA or recruiting transcription factors. 4) LncRNAs serve as a precursor of miRNAs (Zhou et al., 2020). Although dysregulated lncRNAs in human cancer disease are continuously being identified, the biological functions and mechanisms of most lncRNAs in cancer remain unclear. In this study, we focused on the expression and biological roles of HCG18 in BC. HCG18 was identified as one of the 30 upregulated lncRNAs in BC from the analysis of data from two cohorts in the TCGA database (Xu et al., 2017). However, its exact expression profile and biological function in BC were unclear. Therefore, we detected HCG18 expression in BC tissues for the first time, and the results were consistent with the results in the TCGA database. We found that HCG18 is highly expressed in BC tissues and cells. Furthermore, we found that BC patients with high HCG18 expression had worse DMFS and OS, and these results were further confirmed with survival information from the Kaplan–Meier Plotter database. Then, we established stable HCG18^*sh*^/HCG18^*OE*^ BC cells and used both *in vitro* and *in vivo* assays to evaluate HCG18’s biological function. All the results confirmed that HCG18 serves as an oncogene by promoting proliferation and invasion and endowing BC cells with CSPs. HCG18 can also play tumor-promoting roles in many other types of cancers. HCG18 can increase the expression levels of WIPF1 and DNAJB12 and activate the PI3K/AKT axis in gastric cancer cells, thus promoting gastric cancer progression (Liu et al., 2020a; Ma et al., 2020; Niu et al., 2020). HCG18 can contribute to hepatocellular carcinoma progression by increasing CENPM expression (Zou and Sun, 2020). HCG18 can also act as an oncogene in lung adenocarcinoma by enhancing HMMR expression and can accelerate nasopharyngeal carcinoma progression by upregulating CCND1 (Li et al., 2019; Li et al., 2020a). All these achievements indicated that HCG18 may be a potential diagnostic marker and a novel antitumor target for cancer therapy. HCG18 is also involved in some nonneoplastic diseases, such as diabetic peripheral neuropathy, coronary atherosclerotic heart disease, vascular disease, and intervertebral disc degeneration (Li et al., 2018; Wang et al., 2020a; Ren et al., 2021), which means that HCG18 is an important pathogenic factor in human diseases and needs to be further studied.

UBE2O is a large E2 ubiquitin-conjugation enzyme that has both E2 and E3 enzyme properties and activities (Berleth, 1996). Deregulation of UBE2O has been associated with several human diseases, especially cancers. UBE2O could mediate Mxi1 ubiquitination and then promote lung cancer progression and radioresistance (Huang et al., 2020). It has been reported that UBE2O could decrease the stability of MLL in a polyubiquitination-dependent manner, which results in aggressive leukemia (Liang et al., 2017). Our previous study proved that UBE2O could promote BC cell proliferation and EMT and endow BC cells with CSPs through a UBE2O/AMPKα2/mTORC1/MYC positive feedback loop (Liu et al., 2020b). However, even though UBE2O is an important oncoprotein, its regulatory mechanism has not been elaborated in detail. In this study, we found that there was a positive relationship between HCG18 and UBE2O expression in BC cells and tissues. To further investigate this, we performed bioinformatics analysis, luciferase assays, and RIP assays. All the results confirmed that UBE2O was a direct downstream target of miR-103a-3p and that HCG18 could enhance UBE2O mRNA stability by competitively absorbing miR-103a-3p in a ceRNA-dependent manner. Furthermore, we first verified that miR-103a-3p is downregulated in BC tissues and serves as a tumor suppressor in BC. Chang et al. analyzed miRNA expression profiles and their correlation with the prognosis of BC patients in the TCGA database. They reported that miR-103a-3p expression was decreased in BC tissues and contributed to a better prognosis in BC patients (Chang et al., 2016). Our result was in accordance with this previous result and further expanded our knowledge about the regulatory mechanism of UBE2O in BC.

HIF-1α is a crucial regulator of metabolism and a well-characterized oncoprotein in cancer cells (Gilkes, 2013). By modulating the expression of a series of glycolytic enzyme genes, HIF-1α could stimulate angiogenesis, enhance aerobic glycolysis, and mediate metabolic reprogramming, which in turn facilitates BC cell malignant transformation (Wilson, 2011). HIF-1α can also bind to hypoxia response elements (HREs) in the promoter regions of target genes and act as a transcription factor in regulating the expression of multiple cancer-related genes (Lee et al., 2020). The stabilization of HIF-1α is vital for cells in response to oxygen change and can be regulated by PHD and pVHL (Xu et al., 2020). In addition, HIF-1α can also be regulated by other regulators, including noncoding RNAs. Previous studies reported several lncRNAs participating in tumorigenesis and metastasis, such as lncRNA EFNA3, lncRNA BCRT1, and lncRNA HITT, have been proven to be regulated by HIF-1α-mediated transcriptional regulation (Gomez-Maldonado et al., 2015; Wang et al., 2020b; Liang et al., 2020). In our study, we identified HIF-1α as a potential transcription factor of HCG18, and we found four potential HREs in the promoter region of HCG18 in the JASPAR database. Reducing HIF-1α suppressed HCG18 expression in BC cells, and there was a positive correlation between HCG18 and HIF-1α expression in both BC tissues and the GEPIA database. ChIP and dual-luciferase reporter assays further confirmed that HIF-1α could bind to specific HREs in the promoter region of HCG18 and promote HCG18 expression in BC cells. This self-controlled positive feedback loop further highlights the significance of the HCG18/miR-103a-3p/UBE2O/mTORC1-HIF-1α axis in BC.

## Conclusion

Our study confirmed that HCG18 positively regulates UBE2O expression by sponging miR-103a-3p and subsequently mediates the malignant phenotypes of BC cells, thus playing an oncogenic role in BC progression. Our research provides a new theoretical basis for exploring the mechanism of proliferation, invasion, and CSPs in BC. Collectively, our study first demonstrated that the HCG18/miR-103a-3p/UBE2O/mTORC1-HIF-1α axis constitutes a positive feedback loop in promoting proliferation and invasion and enhancing CSPs in BC. HCG18 could become a promising therapeutic target and prognostic predictor for BC.

## Data Availability

The raw data supporting the conclusion of this article will be made available by the authors, without undue reservation.
