# Supplementary material for: Long Noncoding RNA HCG18 Promotes Malignant Phenotypes of Breast Cancer Cells via the HCG18/miR-103a-3p/UBE2O/mTORC1/HIF-1α–Positive Feedback Loop
Source: Front Cell Dev Biol. 2021 Dec 7;9:675082. doi: 10.3389/fcell.2021.675082 (PMC8715259; doi:10.3389/fcell.2021.675082)
Supplement: Supplementary file 1 [file Table1.docx]

| **Supplementary Table 1ⅠPrimers for qRT-PCR** | | |
| --- | --- | --- |
| Name | Sequence | |
| HCG18-F | | GCTAGGTCCTCTACTTTCTG |
| HCG18-R | | CAGAAAGTAGAGGACCTAGC |
| miR-30c-5p-F | | GCCGCTGTAAACATCCTACACT |
| miR-30c-5p-R | | GTGCAGGGTCCGAGGT |
| miR-34a-5p-F | | ACACTCCAGCTGTGACTGGTTGACCAGA |
| miR-34a-5p-R | | CTCAACTGGTGTCGTGGA |
| miR-103a-3p-F | | AGCAGCATTGTACAGGGCTA TGAA |
| miR-103a-3p-R | | TGGTGTCGTGGAGTCG |
| UBE2A-F | | CTCTCTCTGCTCTCAGGTTGGTTC |
| UBE2A-R | | ATTCCACTCAAGCCTTTAGCAG |
| UBE2B-F | | ATTGCAGGGTTGTTTGTCAGTC |
| UBE2B-R | | TGGCACTTAAAATTTGTTAGCC |
| UBE2C-F | GGATTTCTGCCTTCCCTGAA | |
| UBE2C-R | GATAGCAGGGCGTGAGGAAC | |
| UBE2D1-F | ATCCACCTGCTCACTGTTCA | |
| UBE2D1-R | GTGACCATTGTGACCTCAGA | |
| UBE2D3-F | AAAGGATCCATGGCGCTGAAACGGATTAAT | |
| UBE2D3-R | TTTCTCGAGTCACATGGCATACTTCTGAGTCCATTC  CCGAGA | |
| UBE2E1-F | TTTGGATCCATGTCGGATGACGATTCGAGG | |
| UBE2E1-R | TTTCTCGAGTTATGTAGCGTATCTCTTGGTCC | |
| UBE2E2-F | AGGTTACCTTCCGAACAAGAAT | |
| UBE2E2-R | AAATAGTTAAAGCCGGACTCCA | |
| UBE2F-F | ATTGCGGCCGCCTAACGCTAGCAAGTAAACTGA | |
| UBE2F-R | CGCCTCGAGTCATCTGGCATAACGTTTGATGTAGTCATC | |
| UBE2G1-F | ATGAAGGTGGTGTTTTTAAGGC | |
| UBE2G1-R | TCCTCTGGCTTTTCATAACCAT | |
| UBE2H-F | AGTTAGAGTGGACCTACCTGAT | |
| UBE2H-R | ACATCTAGACACACAGTTCCTG | |
| UBE2I-F | GCTTGTTTAAACTACGGATGCT | |
| UBE2I-R | CATTCGGGTGAAATAATGGTGG | |
| UBE2J1-F | GCTTGTTTAAACTACGGATGCT | |
| UBE2J1-R | CATTCGGGTGAAATAATGGTGG | |
| UBE2J2-F | GAGCAGCACCAGCAGTAAGAGG | |
| UBE2J2-R | GGCTCGGCACAGATGTAAGGC | |
| UBE2L3-F | CAAACCACCGAAGATCACATTT | |
| UBE2L3-R | ATTACTTGGTCGGTTTTGGTTG | |
| UBE2L6-F | AGCTGGAGGATCTTCAGAAGAA | |
| UBE2L6-R | TGGTTGTGAATTTGATCATGGG | |
| UBE2M-F | AAAGGATCCATGATCAAGCTGTTCTCGCTGAAG | |
| UBE2M-R | TTTGCGGCCGCTTATTTCAGGCAGCGCTCAAAG | |
| UBE2N-F | CCTTTGAGGGAGGGACTTTTAA | |
| UBE2N-R | TTAACAAGGCCTGGATCGATAG | |
| UBE2O-F | GAATCCAAAACCAAGAGCGAAG | |
| UBE2O-R | TCATCTCTGCCTTCTTTTAGCA | |
| UBE2Q1-F | GTCAAACTCCTCAAAGTTGACC | |
| UBE2Q1-R | TAAGTAGAATGAAGTCGGCTCC | |
| UBE2Q2-F | GGAAGAAGAAGAAGAGATGGCT | |
| UBE2Q2-R | TTCATCCTCTGACTTTTTCCCA | |
| UBE2R2-F | ATGCATTTCGATTCTTCATCCG | |
| UBE2R2-R | CCTGAACATAACTGAAGCATCG | |
| UBE2S-F | CATATGCTGGAGGTCTGTTCC | |
| UBE2S-R | AAGATCTTGGTCAGGAAGTAGC | |
| UBE2T-F | CCAAACATTGATTCTGCTGGAA | |
| UBE2T-R | TCAGCCTTTTGTTTCTGTCTTG | |
| UBE2U-F | TGGCAGGGTTTAGTCTTCCAACTG | |
| UBE2U-R | CTATACAGGGCTGACCAGTGTGTG | |
| UBE2Z-F | TCTGTGACATGATGGAAGGAAA | |
| UBE2Z-R | CAAAAGGGTCCTGCATAGTTTG | |
| GAPDH-F | GGAGCGAGATCCCTCCAAAAT | |
| GAPDH-R | GGCTGTTGTCATACTTCTCATGG | |
| U6-F | CTCGCTTCGGCAGCACA | |
| U6-R | AACGCTTCACGAATTTGCGT | |
| 18S rRNA-F | GTAACCCGTTGAACCCCATT | |
| 18S rRNA-R | CCATCCAATCGGTAGTAGCG | |
