# Supplementary figures and images for: Long Noncoding RNA HCG18 Promotes Malignant Phenotypes of Breast Cancer Cells via the HCG18/miR-103a-3p/UBE2O/mTORC1/HIF-1α–Positive Feedback Loop
Source: Front Cell Dev Biol. 2021 Dec 7;9:675082. doi: 10.3389/fcell.2021.675082 (PMC8715259; doi:10.3389/fcell.2021.675082)

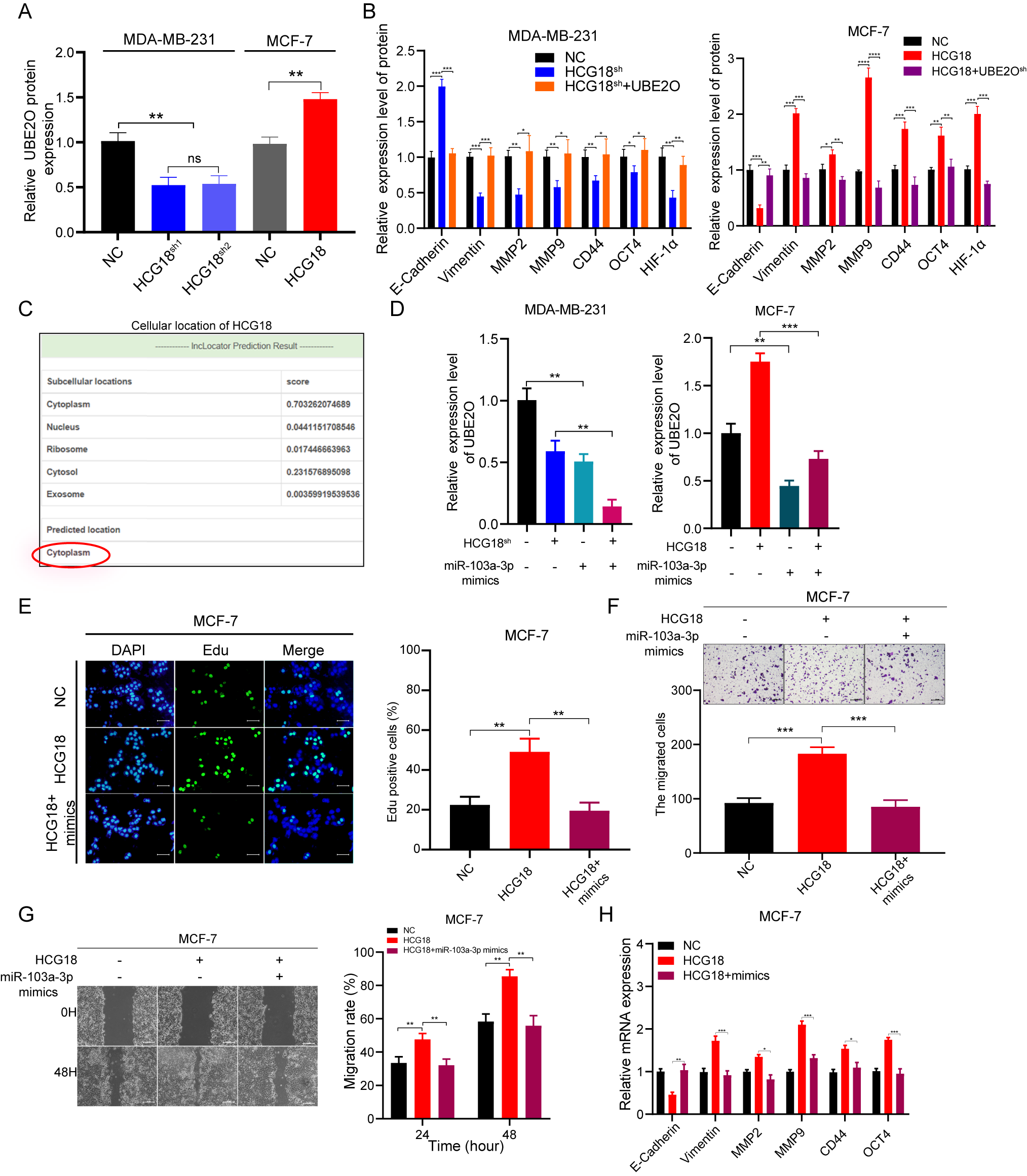

Supplement: Supplementary file 2 [file Image2.tif]

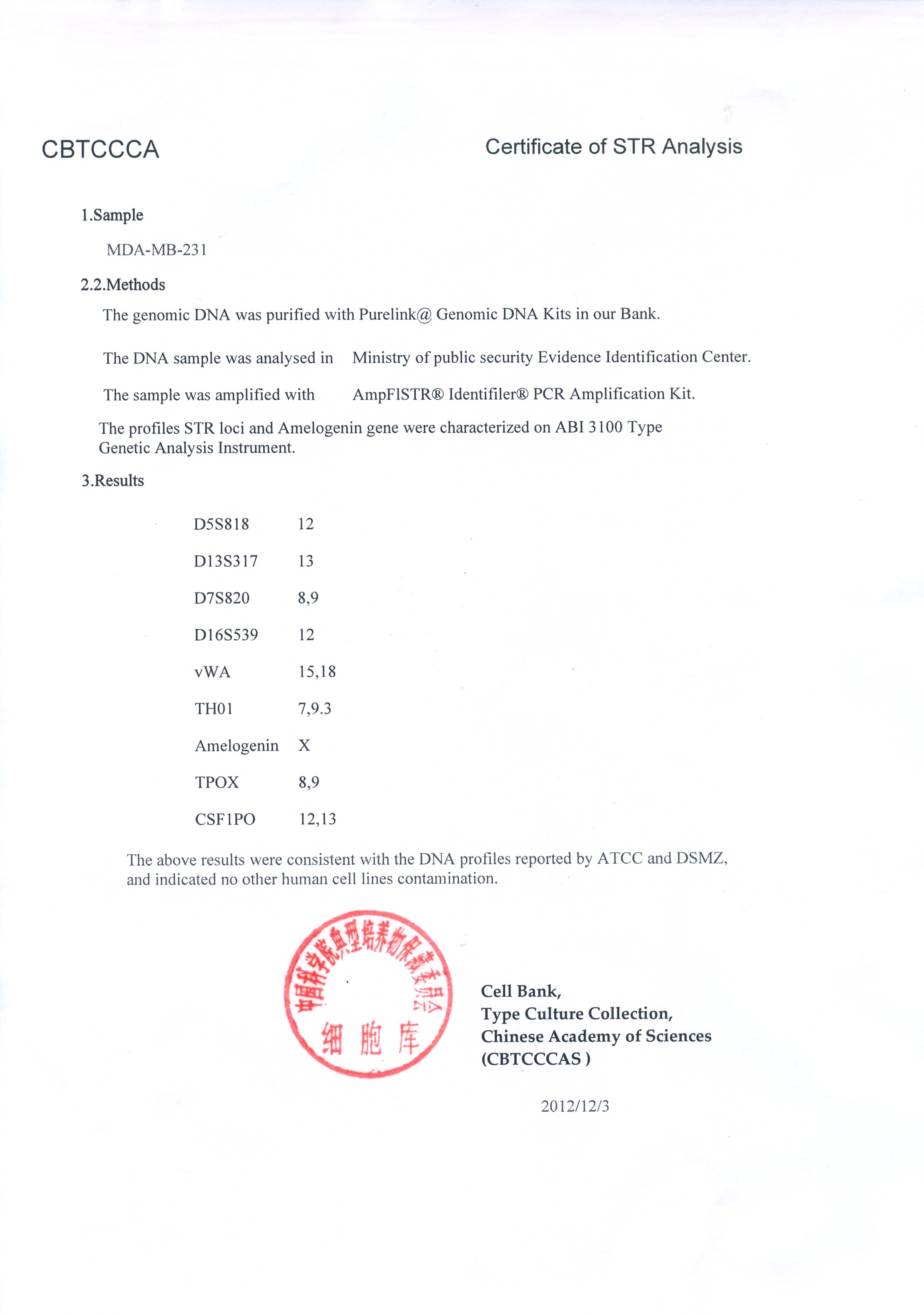

Supplement: Supplementary file 3 [file Image2.JPEG]

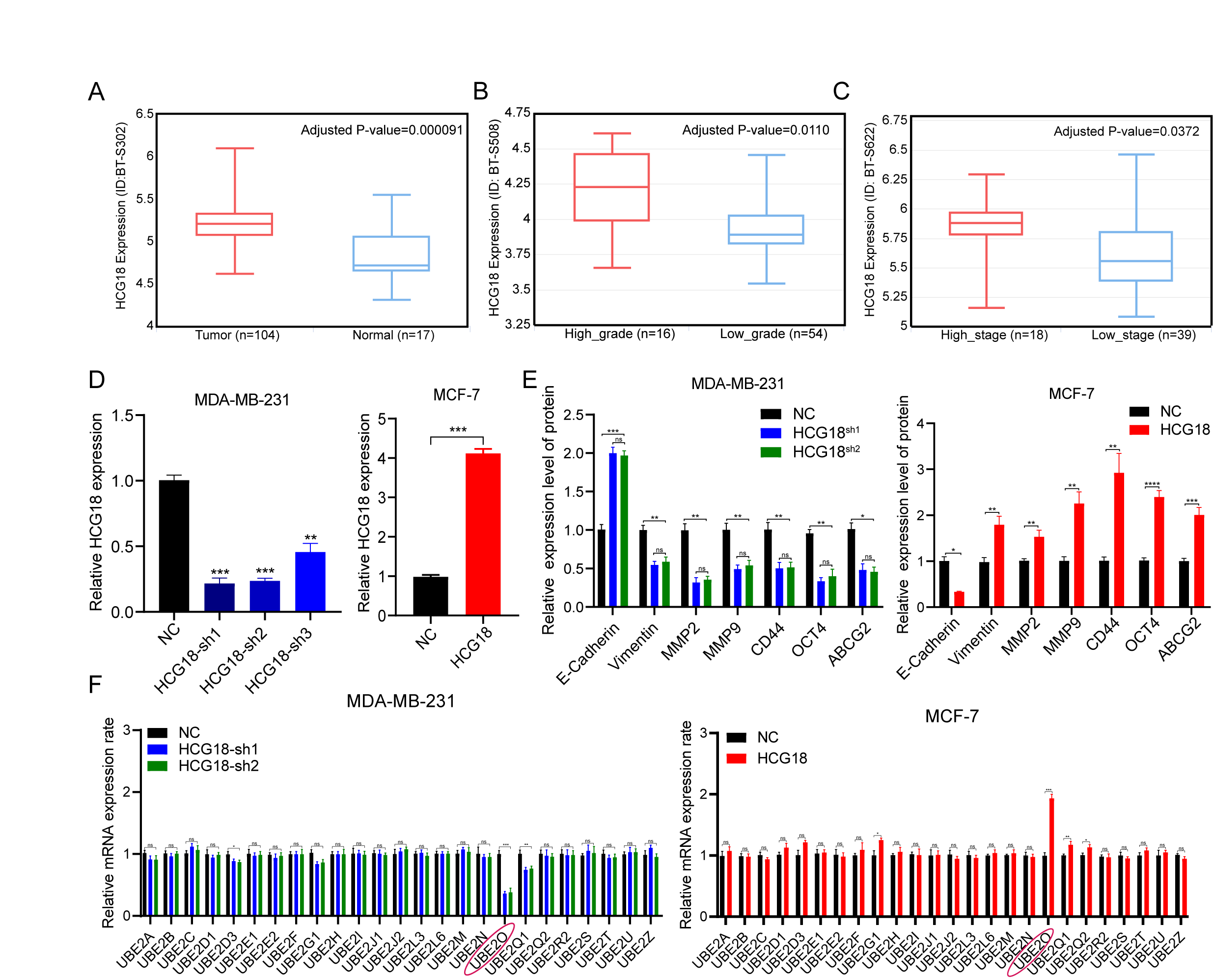

Supplement: Supplementary file 4 [file Image1.tif]
