# Supplementary material for: Long Noncoding RNA HCG18 Promotes Malignant Phenotypes of Breast Cancer Cells via the HCG18/miR-103a-3p/UBE2O/mTORC1/HIF-1α–Positive Feedback Loop
Source: Front Cell Dev Biol. 2021 Dec 7;9:675082. doi: 10.3389/fcell.2021.675082 (PMC8715259; doi:10.3389/fcell.2021.675082)
Supplement: Supplementary file 5 [file Table2.docx]

| **Supplementary Table 2ⅠAntibodies for western blotting assays** | | |
| --- | --- | --- |
| Name | Manufacturer/Nationality | Catalogue Number |
| CD44 | CST/USA | 3570S |
| E-Cadherin | CST/USA | 14472 |
| HIF-1α | CST/USA | 36169S |
| MMP2 | CST/USA | 40994 |
| MMP9 | CST/USA | 13667 |
| OCT4 | CST/USA | 2750 |
| Vimentin | CST/USA | 5741 |
| UBE2O | CST/USA | 83393S |
|  |  |  |
