# Supplementary material for: Long Noncoding RNA HCG18 Promotes Malignant Phenotypes of Breast Cancer Cells via the HCG18/miR-103a-3p/UBE2O/mTORC1/HIF-1α–Positive Feedback Loop
Source: Front Cell Dev Biol. 2021 Dec 7;9:675082. doi: 10.3389/fcell.2021.675082 (PMC8715259; doi:10.3389/fcell.2021.675082)
Supplement: Supplementary file 6 [file Table3.docx]

| **Supplementary Table 3ⅠsiRNA for target gene** | | |
| --- | --- | --- |
| Name | Sequence | |
| si-HIF-1α#1: | | 5’CCGGCCAGTTATGATTGTGAAGTTACTCGAGTAACTTCACAATCATAACTGGTTTTT3’  5’CCGGCCGCTGGAGACACAATCATATCTCGAGATATGATTGTGTCTCCAGCGGTTTTT3’  5′UUGGCUUCAGUCCUGUUCAUCAG3′  5′TTGGCTTCAGTCCTGTTCATCAG3′  5’CCGGCGATGATTCCTATGGCTTCTACTCGAGTAGAAGCCATAGGAATCATCGTTTTT3’ |
|  | |  |
| si-HIF-1α#2: | |  |
|  | |  |
| sh-HCG18#1: | |  |
| sh-HCG18#2: | |  |
| sh-UBE2O: | |  |
|  | |  |
